# The Homœopathic Physician’s Visiting List and Pocket Repertory

**Published:** 1881-12

**Authors:** 


The Homœopathic Physicians' Visiting List and Pocket Repertory. By Robert Faulkner, M. D., 2d edition, price $2.00, Bœricke & Tafel: New York and Philadelphia.

This "pocket-book" contains records for vaccinations, accouchements, deaths, and for ordinary routine visits, having places for marking visits made and medicine given. An obstetric calendar, table of pulse, antidotes for poisons, etc., are not forgotten. It is very complete and very neatly gotten up.

The repertory consists of some ninety pages arranged alphabetically, as it should be. The repertory being so small, must of course be a mere epitome; yet it will furnish hints that will assist the physician.

While some of the indications are rather vague and indefinite, others are positively erroneous and misleading. For instance, under "croup," we read: "*Aconite* should always be given at the first, unless some other remedy is perfectly indicated." We would suggest that *Aconite* should NEVER be given unless perfectly indicated, no matter what "stage" is being treated! *Aconite* is the most abused remedy in our materia medica.

Again, under "Typhoid," we read: "Typhoid fever may be cut off in the beginning by *Gels.* in water, given every fifteen minutes, until profuse sweating is produced, and continued thereafter every half-hour until all pains and aching are gone." We hope none of our readers will ever try this "cutting short" method.

